# Bioinformatics analysis of human kallikrein 5 (*KLK5*) expression in metaplastic triple‐negative breast cancer

**DOI:** 10.1002/cai2.96

**Published:** 2023-10-15

**Authors:** Yue Song, Guiying Bai, Xiaoqing Li, Liyan Zhou, Yiran Si, Xiaohui Liu, Yilin Deng, Yehui Shi

**Affiliations:** ^1^ Department of Phase I Clinical Trial Tianjin Medical University Cancer Institute and Hospital Tianjin China; ^2^ Medical Oncology Department of Breast Cancer Tianjin Medical University Cancer Institute and Hospital Tianjin China; ^3^ National Clinical Research Center for Cancer Tianjin China

**Keywords:** bioinformatics analysis, differentially expressed genes, EMT, *KLK5*, metaplastic breast carcinoma

## Abstract

**Background:**

Metaplastic breast carcinoma (MBC) is a rare breast cancer subtype; most cases are triple‐negative breast cancers (TNBCs) and are poorly responsive to conventional systemic therapy. Few potential diagnostic and prognostic markers for distinguishing between metaplastic TNBC and nonmetaplastic TNBC have been discovered. We performed bioinformatic analysis to explore the underlying mechanism by which metaplastic TNBC differs from nonmetaplastic TNBC and provides potential pathogenic genes of metaplastic TNBC.

**Methods:**

Differentially expressed genes (DEGs) in metaplastic tumors and nonmetaplastic tumors from TNBC patients were screened using GSE165407. The GSE76275 data set and The Cancer Genome Atlas (TCGA) database were used to screen DEGs in TNBC and non‐TNBC. Metascape and DAVID were used for the Kyoto Encyclopedia of Genes and Genomes (KEGG) enrichment analysis and Gene Ontology (GO) analysis of DEGs. Online databases, including UALCAN, GEPIA, HPA, Breast Cancer Gene‐Expression Miner, and quantitative PCR and western blot, were used to examine *KLK5* messenger RNA and protein expression in breast cancer. Analysis of *KLK5*‑associated genes was performed with TCGA data, and the LinkedOmics database was used to detect the genes co‐expressed with *KLK5*. STRING (Search Tool for the Retrieval of Interacting Genes) and Cytoscape were used to screen for hub genes. Kaplan‑Meier plotter was used for survival analysis.

**Results:**

*KLK5* was identified among the DEGs in nonmetaplastic TNBC and metaplastic TNBC. The *KLK5* gene was overexpressed in nonmetaplastic TNBC but downregulated in metaplastic TNBC. KEGG and GO analyses revealed that epithelial‐to‐mesenchymal transition was a pathogenic mechanism in metaplastic TNBC and an important pathway by which *KLK5* and its associated genes *DSG1* and *DSG3* influence metaplastic TNBC progression. Prognosis analysis showed that only low expression of *KLK5* in metaplastic TNBC had clinical significance.

**Conclusion:**

Our research indicated that *KLK5* may be a pivotal molecule with a key role in the mechanism of tumorigenesis in metaplastic TNBC.

## Graphical abstract

Metaplastic breast carcinoma is a rare breast cancer subtype; most of which belongs to triple‐negative breast cancer(TNBC) and is poorly responsive to contemporary systemic therapy. Kallikrein 5 (*KLK5*) was overexpressed in nonmetaplastic TNBC but downregulated in metaplastic TNBC, which was detected by bioinformatics analysis. *KLK5* is considered to be a driver gene in metaplastic TNBC because of its low expression and is associated with the aggressiveness of metaplastic TNBC by promoting epithelial‐to‐mesenchymal transition development.

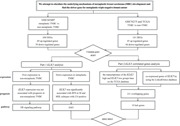

**Abbreviations**
DEGs: differentially expressed genes
EMT: epithelial‐to‐mesenchymal transition
GEO: Gene Expression Omnibus
GO: Gene Ontology
IM: immunomodulatory
KEGG: Kyoto Encyclopedia of Genes and Genomes
LN: lymph node
M: mesenchymal
MBC: metaplastic breast carcinoma
MSL: mesenchymal stem‐like
RFS: relapse‐free survival
TCGA: The Cancer Genome Atlas
TNBC: triple‐negative breast cancer

## 1 INTRODUCTION

Metaplastic breast carcinoma (MBC) represents a morphologically heterogeneous group of invasive breast cancers characterized by the presence of malignant epithelial cells showing features of myoepithelial differentiation [1]. Most MBCs display a triple‐negative phenotype (lack of expression of estrogen receptor [ER], progesterone receptor [PR], and HER2) and are classified as the basal‐like or claudin‐low molecular subtype. However, MBCs have a more aggressive clinical behavior than these tumors, and fewer effective therapies are available [2].

Few studies have compared MBC to other types of breast cancer. Some reports showed that EGFR and pan‐cytokeratin expression have prognostic implications in MBC [3]. Several studies have focused on prognostic factors for relapsed metastatic MBC. Next‐generation sequencing was used to identify clinically meaningful alterations and guide targeted treatment in 20 metastatic MBCs [4]. Another study characterized the molecular profile and tumor evolution in primary‐relapse MBC samples compared with recurrence/progression samples [5]. Some studies analyzed one subtype of MBC to identify important markers that help distinguish it from other subtypes of MBC [6]. However, no studies have analyzed the differentially expressed genes (DEGs) between metaplastic triple‐negative breast cancer (TNBC) and nonmetaplastic TNBC.

While MBC has been shown to consistently harbor a TNBC immunophenotype and expression profile, it exhibits lower response rates to neo(adjuvant) systemic treatment and has higher rates of disease progression, recurrence, and mortality than TNBC [7, 8]. Because of the limited understanding of its pathogenesis, patients with MBC receive similar treatments as TNBC patients, but MBC is typically chemoresistant. In contrast to other subtypes, MBC, especially with spindle cell metaplasia, frequently displays stem cell‐like and epithelial‐to‐mesenchymal transition (EMT) characteristics [9, 10]. MBC also harbors mutated genes involved in the phosphatidylinositol 3‐kinase signaling pathway and DNA damage repair mechanisms but it is not driven by a highly expressed gene fusion or a highly recurrent mutation affecting previously described cancer genes [11, 12, 13, 14]. Further studies are needed to explore the specific mechanisms of MBC and identify key genes to establish targeted therapy.

MBC exhibits characteristics of early relapse, high invasion and metastasis, lack of standard effective treatment, and poor prognosis. Thus, exploration of DEGs in metaplastic TNBC compared with nonmetaplastic TNBC may provide insights into the mechanism of metaplastic TNBC and identify prognostic markers of metaplastic TNBC. In this study, we investigated DEGs by searching public data sets and performing bioinformatics analysis with the aim of identifying key genes involved in the tumorigenesis and progression of metaplastic TNBC that can be used to distinguish metaplastic TNBC from nonmetaplastic TNBC.

## 2 METHODS

### 2.1 Microarray data

GSE76275 and GSE165407 were obtained from the Gene Expression Omnibus (GEO, http://www.ncbi.nlm.nih.gov/geo), which is a free public repository for data storage, including microarray data and next‐generation sequencing. The GSE76275 data set was obtained from the Affymetrix Human Genome U133 Plus 2.0 Array, consisting of 198 TNBC tissue samples and 67 non‐TNBC tissue samples. The GSE165407 data set was obtained from Illumina HiSeq. 2000 (*Homo sapiens*), consisting of eight treatment‐naïve metaplastic tumors and 20 treatment‐naïve nonmetaplastic tumors from TNBC patients. Whole‐transcriptome sequencing data of 122 TNBC and 655 non‐TNBC samples were downloaded from The Cancer Genome Atla (TCGA) database (http://cancergenome.nih.gov).

### 2.2 Screening for DEGs

The *p* values and Benjamini and Hochberg false discovery rate (FDR) were applied to provide a balance between the discovery of statistically significant genes and limitations of false positives. *p* < 0.001 and |fold change (FC)| ≥ 1.5 were set as the thresholds for identifying DEGs in GEO databases. RNA‐sequencing (RNAseq) data were downloaded from TCGA database. Differential messenger RNA (mRNA) expression was analyzed in TNBC samples and non‐TNBC samples with the R software with the Limma package. The screening criteria used in TCGA were as follows: *p* < 0.001 and |FC| ≥ 2.

The kallikrein 5 (*KLK5*)‐low and *KLK5*‐high groups were categorized using the median values of the *KLK5* transcript in the RNAseq data from TCGA. The Limma package in R 3.3.3 was used to screen DEGs between the *KLK5*‐high and *KLK5*‐low groups in breast cancer. *p* < 0.001 and |log 1.2 FC| ≥ 1.5 were used as cut‐off values for identifying DEGs.

### 2.3 Gene Ontology (GO) functional enrichment and Kyoto Encyclopedia of Genes and Genomes (KEGG) analysis

Metascape [15] (http://metascape.org/gp/index.html#/main/step1) is an online biological information database that integrates biological data and analysis tools and was used to perform functional enrichment analysis for the DEGs. The Database for Annotation, Visualization and Integrated Discover (DAVID) is another online tool (http://david.abcc.ncifcrf.gov) [16, 17] that provides a comprehensive set of high‐throughput functional gene analyses to understand the enrichment of KEGG for DEGs and GO enrichment analysis for co‐expression DEGs. KEGG is a database resource for understanding high‐level functions and biological systems from large‐scale molecular data sets generated by high‐throughput experimental technologies. GO is a major bioinformatics tool to annotate genes and analyze the biological processes of these genes. A *p* < 0.05 was considered statistically significant.

### 2.4 Survival analysis

The prognostic values of *KLK5* in TNBC were analyzed using the Kaplan‑Meier plotter database [18], an online software for survival analysis. Kaplan‑Meier survival plots were generated and hazard ratio, 95% confidence intervals, and log‐rank *p* value were displayed. A *p* value of <0.05 was regarded as statistically significant.

### 2.5 Analysis of target gene expression using databases

UALCAN (http://ualcan.path.uab.edu/), a portal for facilitating tumor subgroup gene expression and survival analyses, provides easy access to publicly available cancer transcriptome data including TCGA [19]. GEPIA is a new web‐based tool that provides differential expression analysis of tissues using TCGA [20]. These two databases were used to analyze the expression level of *KLK5* in subtypes of breast cancer. Statistical analysis of the comparison between TNBC and normal groups was performed and log‐rank *p* value was observed in the database. HPA [21] (https://www.proteinatlas.org/) contains the human transcriptomic and proteomic data in cells, tissues, and organs from normal or diseased cases using RNAseq analysis and immunohistochemistry. *KLK5* expression was analyzed in various breast cancer cell lines.

### 2.6 LinkedOmics database analysis

A new and unique tool LinkedOmics (http://www.linkedomics.org) [22] was used to determine *KLK5* co‐expression in breast cancer. *KLK5* co‐expression genes in breast cancer were determined by analyzing mRNA sequencing data from TCGA database, and Pearson's correlation coefficient was calculated to analyze the data. Volcano plots were used to display the results.

### 2.7 Breast cancer gene expression analysis

The expression of *KLK5* mRNA in different subtypes of breast cancer was analyzed using the Breast Cancer Gene‐Expression Miner (bcGenExMinerv4.5; http://bcgenex.centregauducheaufr/BC-GEM) [23].

### 2.8 Integration of protein–protein interaction (PPI) networks and expression analysis of hub genes

The PPI network was analyzed using the Search Tool for the Retrieval of Interacting Genes (STRING; http://string-db.org) (version 10.0) online database [24]. DEGs were analyzed using the STRING database. Cytoscape (version 3.4.0) is an open‐source bioinformatics software platform for visualizing molecular interaction networks. The cytoHubba of Cytoscape is an APP for screening for hub genes by selecting with degrees ≥10.

### 2.9 RNA extraction and quantitative PCR (qPCR)

TRIzol reagent (Invitrogen/Thermo Fisher Scientific) was used to extract RNA from MCF‐10A, HS578T, and MDA‐MB‐231 cell lines (Cellcook). Equal amounts of RNA were converted into complementary DNA using the Prime Script RT Reagent Kit (Takara Bio Inc.). qPCR was performed using a Reverse Transcription System (Promega) on a CFX96 Real‐Time PCR Detection System (Bio‐Rad Laboratories) following the instructions of the manufacturer. Each sample was analyzed in triplicate. Relative expression was calculated using the $2^{-\Delta \Delta C_{q}}$ method [25]. The PCR conditions were as follows: 40 cycles of 95°C for 30 s, 56°C for 30 s, 72°C for 90 s, and a final extension at 72°C for 5 min. The primer sequences were as follows: *KLK5*, forward: AGAGCATGTTCTCGCCAACAA; reverse: TGGGTGTGCATATCGCAGTC.

### 2.10 Western blot analysis

Tumor tissues were lysed in RIPA buffer supplemented with a proteinase inhibitor cocktail (Sigma). Equal amounts of protein (20 µg) were separated by sodium dodecyl sulfate‐polyacrylamide gel electrophoresis gels and transferred to a polyvinylidene fluoride membrane. After blocking in milk, the membranes were incubated overnight at 4°C with primary antibodies against *KLK5* (1:1000; Abcam; ab176299). The next day, the membranes were incubated with secondary antibodies. Specific protein bands were visualized using an Enhanced Chemiluminescence Detection Reagent (Pierce).

### 2.11 Statistical analysis

Analyses were performed using GraphPad software. The PCR and western blot results were derived from at least three independent experiments. Data are shown as mean ± SD. Differences between groups were estimated using unpaired Student's *t*‐test or analysis of variance test. A two‐tailed value of *p* < 0.05 was considered statistically significant. Other statistical results were provided automatically by the database online software.

## 3 RESULTS

### 3.1 Identification of DEGs between metaplastic TNBC and nonmetaplastic TNBC samples

**Figure 1:**
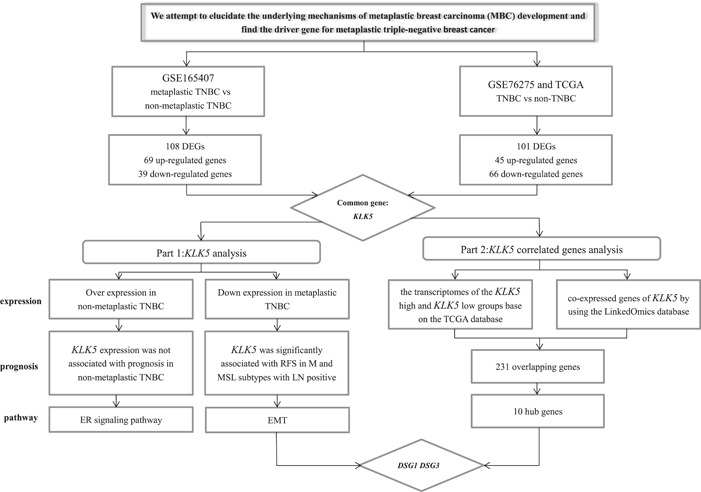
Flowchart of the study. DEG, differentially expressed gene; EMT, epithelial‐to‐mesenchymal transition; ER, estrogen receptor; *KLK5*, kallikrein 5; LN, lymph node; TCGA, The Cancer Genome Atlas; TNBC, triple‐negative breast cancer.

**Figure 2:**
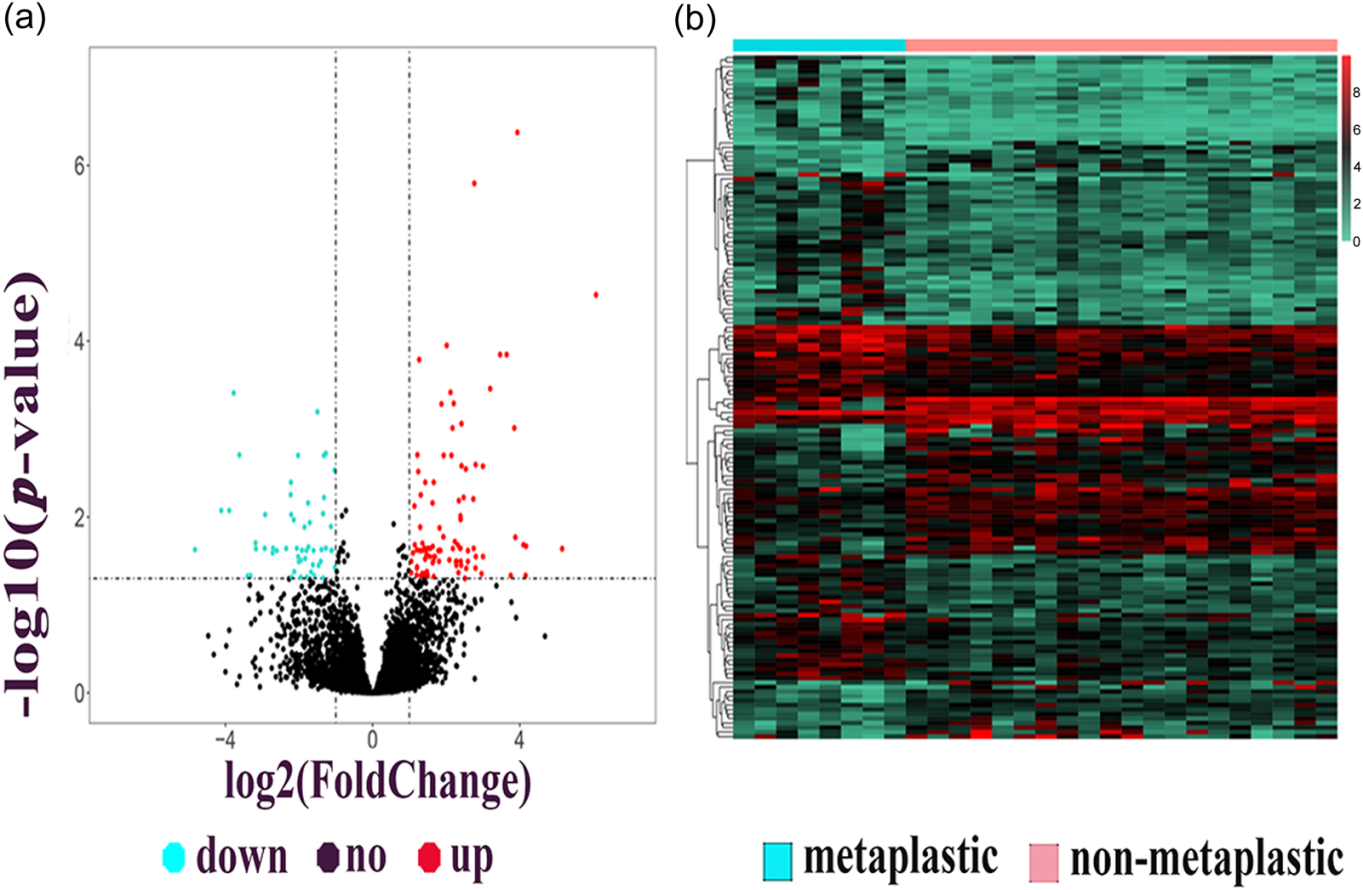
Volcano plot and heatmap of GSE165407. (a) Differentially expressed genes (DEGs) were screened by the following criteria: Log fold change (FC) ≥ 1.5, *p* ≤ 0.001. The black dots represent genes that are not differentially expressed between metaplastic triple‐negative breast cancer (TNBC) and nonmetaplastic TNBC samples, and the red dots and green dots represent the upregulated and downregulated genes in metaplastic TNBC samples, respectively. (b) Heatmap of DEGs in GSE165407.

As most MBCs display a triple‐negative phenotype, we aimed to identify the DEGs in metaplastic TNBC and nonmetaplastic TNBC. The flowchart for this study is shown in Figure 1. We performed differential expression analysis between metaplastic TNBC and nonmetaplastic TNBC using the GEO data set GSE165407. A total of 108 DEGs, including 69 upregulated genes and 39 downregulated genes, were identified by screening genes with |log 2 FC| larger than 1.5 and *p* < 0.001 (Supporting Information: Table S1). Volcano plots and heatmaps of the screened genes from GSE165407 were generated using R with the pheatmap package (Figure 2a,b).

**Figure 3:**
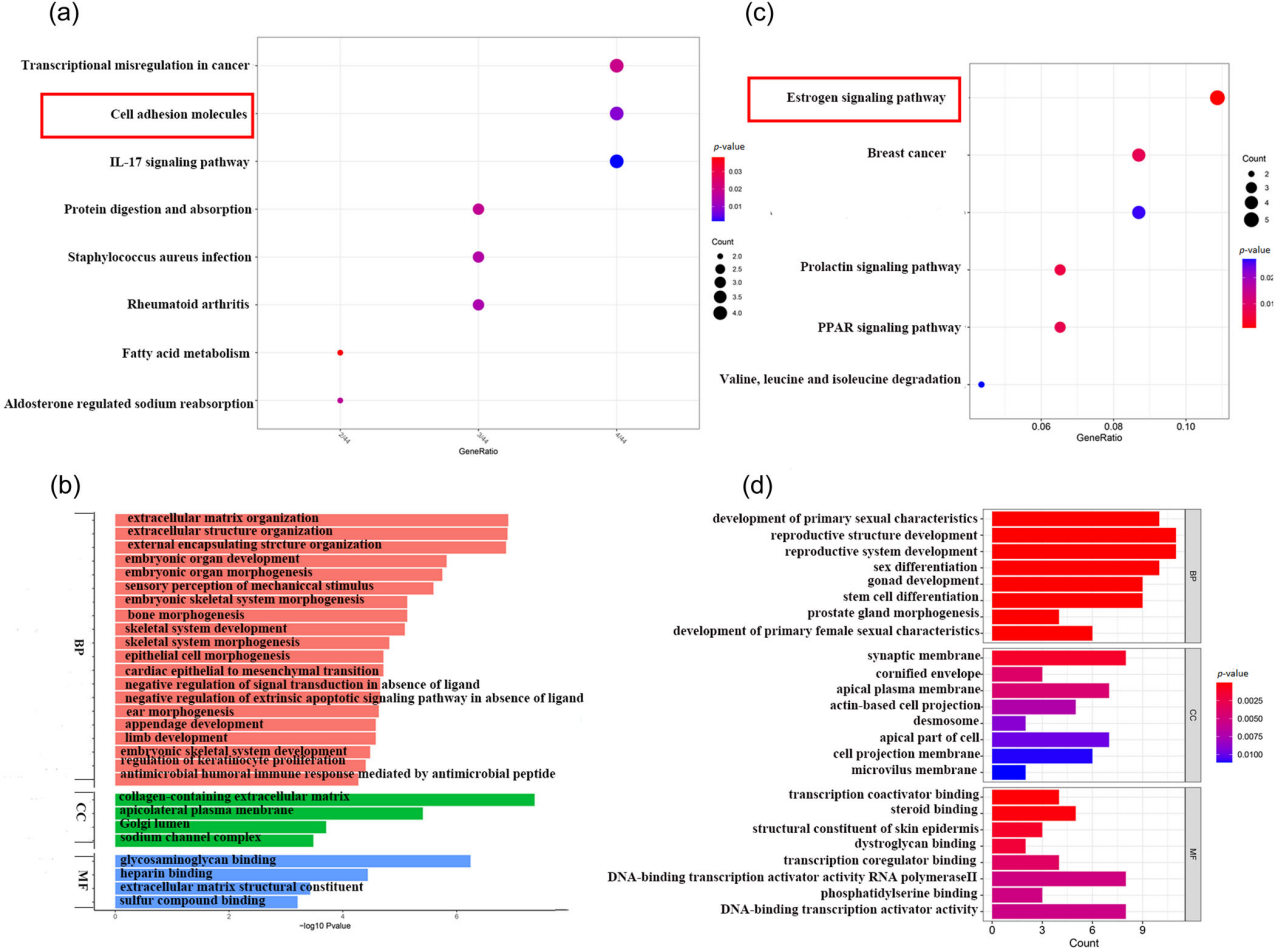
Kyoto Encyclopedia of Genes and Genomes (KEGG) pathway enrichment analysis and Gene Ontology (GO) functions of differentially expressed gene (DEGs). (a) Enriched KEGG pathway analysis of the DEGs in GSE165407. (b) GO analysis of the DEGs in GSE165407. (c) Enriched KEGG pathway analysis of the co‐expression DEGs in GSE76275 and TCGA. (d) GO analysis of the co‐expression DEGs in GSE76275 and TCGA. BP, biological process; CC, cellular component; IL, interleukin; MF, molecular function.

KEGG pathway enrichment analysis of the DEGs in metaplastic TNBC showed several enriched pathways, including the interleukin‐17 signaling pathway, cell adhesion molecules, and transcriptional misregulation in cancer (Figure 3a). The enriched GO terms were mainly associated with extracellular matrix organization, extracellular structure organization, external encapsulating structure organization, collagen‐containing extracellular matrix, and glycosaminoglycan binding (Figure 3b). Compared with nonmetaplastic TNBC, metaplastic TNBC showed an enrichment of genes related to EMT.

### 3.2 The *KLK5* gene is a significant DEG in metaplastic TNBC and nonmetaplastic TNBC

We next compared the significant DEGs in metaplastic TNBC versus nonmetaplastic TNBC and TNBC versus non‐TNBC. We first analyzed DEGs of TNBC and non‐TNBC both from TCGA data sets and from the GSE76275 data set downloaded from the GEO database. A total of 111 genes were found to be significantly differentially expressed in TNBC and non‐TNBC samples, including 45 upregulated and 66 downregulated genes (Supporting Information: Table S2). KEGG enrichment analysis revealed that the estrogen signaling pathway was the most enriched TNBC‐related pathway. GO analysis indicated that the major molecular function of the DEGs was the regulation of transcription (Figure 3c,d). The difference in pathway enrichment may be the main reason for the higher malignancy of metaplastic TNBC compared with nonmetaplastic TNBC.

The *KLK5* gene was the common DEG in the GSE76275, TCGA, and GSE165407 analyses. Notably, *KLK5* showed different expression patterns in nonmetaplastic TNBC and metaplastic TNBC. As shown in Supporting Information: Table S3, *KLK5* was overexpressed in nonmetaplastic TNBC but downregulated in metaplastic TNBC, which suggested that *KLK5* might be a pivotal molecule that plays a different role in the mechanism of tumorigenesis in metaplastic TNBC and nonmetaplastic TNBC. KEGG pathway analysis indicated that *KLK5* is involved in the EMT process of metaplastic TNBC, a key phenotype of this tumor type, while *KLK5* did not show an important molecular function in nonmetaplastic TNBC. Therefore, we speculated that *KLK5* might mediate the EMT process of metaplastic TNBC through a different mechanism than that in nonmetaplastic TNBC and thus promote metaplastic TNBC progression.

### 3.3 *KLK5* expression in breast cancer

**Figure 4:**
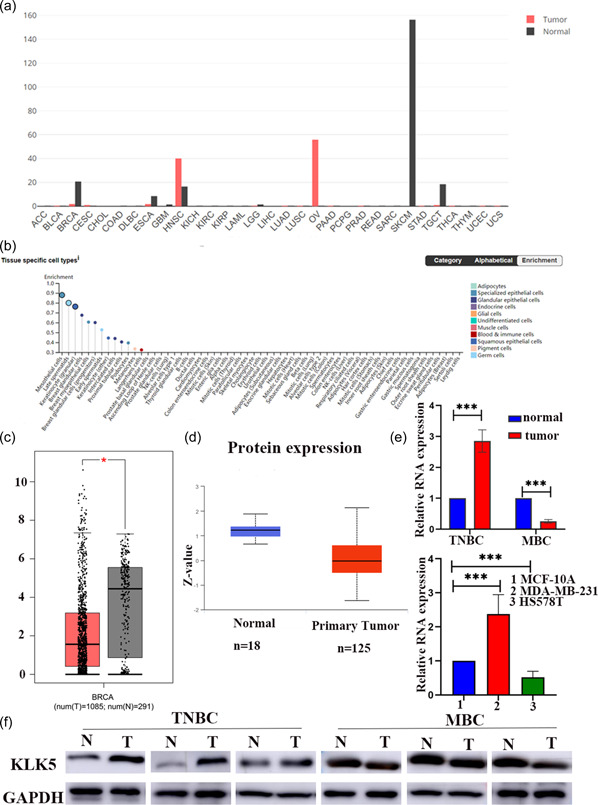
The expression of kallikrein 5 (*KLK5*) in breast cancer. (a) *KLK5* expression in various cancer tissues and normal tissues was analyzed by GEPIA. (b) *KLK5* expression in breast tissue cell types was analyzed by HPA. (c) *KLK5* expression in breast cancer tissues and normal tissues was analyzed by GEPIA. (d) The protein expression of *KLK5* in primary breast cancer and normal tissues was analyzed by the UALCAN cancer database. (e) The messenger RNA expression of *KLK5* was detected by PCR in triple‐negative breast cancer (TNBC) and metaplastic breast carcinoma (MBC) tissues and the MCF‐10A breast cell lines, HS578T metaplastic‐like breast cancer cell line, and MDA‐MB‐231 TNBC cell line. (f) The protein expression of *KLK5* was detected by western blot in TNBC and MBC tissues. GAPDH, glyceraldehyde 3‐phosphate dehydrogenase.

We next analyzed the expression of *KLK5* in breast cancer using different databases. We found that *KLK5* was markedly expressed in breast cancers using GEPIA (Figure 4a). Analysis of the HPA database showed that *KLK5* expression was mainly distributed in breast tissue cell types, including breast glandular cells, breast myoepithelial cells, and breast glandular cells (Figure 4b). *KLK5* mRNA levels were significantly downregulated in breast cancer compared with normal tissues in GEPIA, and *KLK5* protein expression was consistent with the RNA levels in breast cancer in UALAN (Figure 4c,d). We performed PCR and western blot for *KLK5* in TNBC and MBC tissues and cell lines. The mRNA and protein levels of *KLK5* in TNBC and MBC were consistent with the bioinformatic results (Figure 4e,f).

### 3.4 The relationship between *KLK5* expression and clinical indicators in breast cancer patients

**Figure 5:**
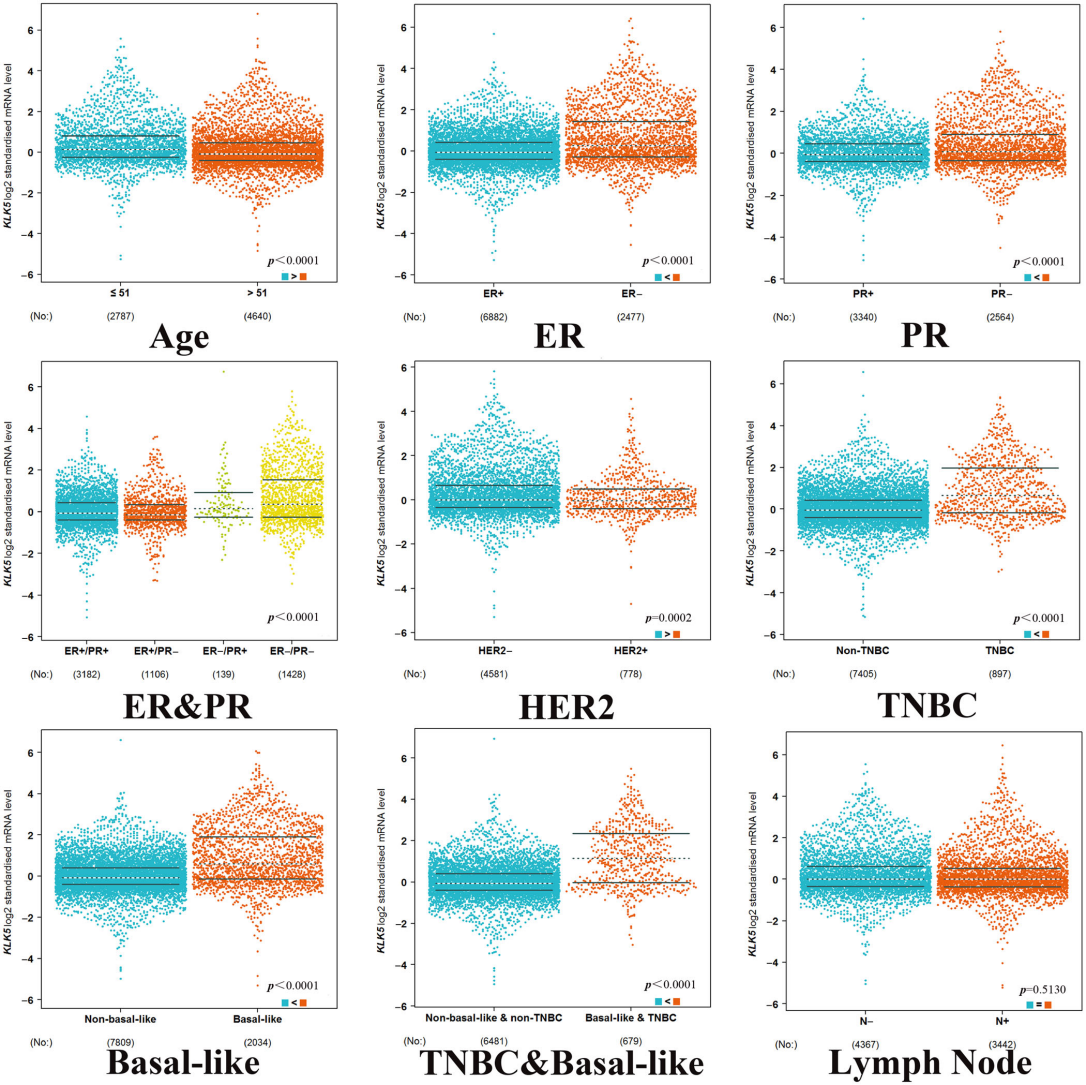
Box plot revealing the relationship between kallikrein 5 (*KLK5*) expression and different clinical indicators. Relationship of *KLK5* expression with the following clinical indicators: age, estrogen receptor (ER), progesterone receptor (PR), ER and PR, HER‐2, basal‐like status, triple‐negative status, basal‐like and triple‐negative status, and nodal status.

We next examined the clinical significance of *KLK5* expression. We next compared *KLK5* expression among subgroups of breast cancer patients using the bc‐GenExMiner online tool. As shown in Figure 5, the expression of *KLK5* was significantly higher in the ≤51 year group than in the >51 year group (Figure 5). *KLK5* expression was higher in ER‐ and/or PR‐negative patients and HER‐2 status was positively associated with *KLK5* expression. Breast cancer patients with basal‐like and TNBC subtypes expressed higher *KLK5* levels, which was consistent with our bioinformatic analysis results. We thus speculated that the ER and PR status has a more important influence on *KLK5* overexpression in TNBC than that of HER2, which is consistent with the KEGG results that the ER signaling pathway is the most enriched pathway in TNBC. However, ER and PR status is not related to *KLK5* downregulation in metaplastic TNBC. A deeper exploration of its function in metaplastic TNBC is required. Breast cancer patients with positive or negative nodal status showed no significant difference in *KLK5* expression.

### 3.5 *KLK5* prognostic value in breast cancer

**Figure 6:**
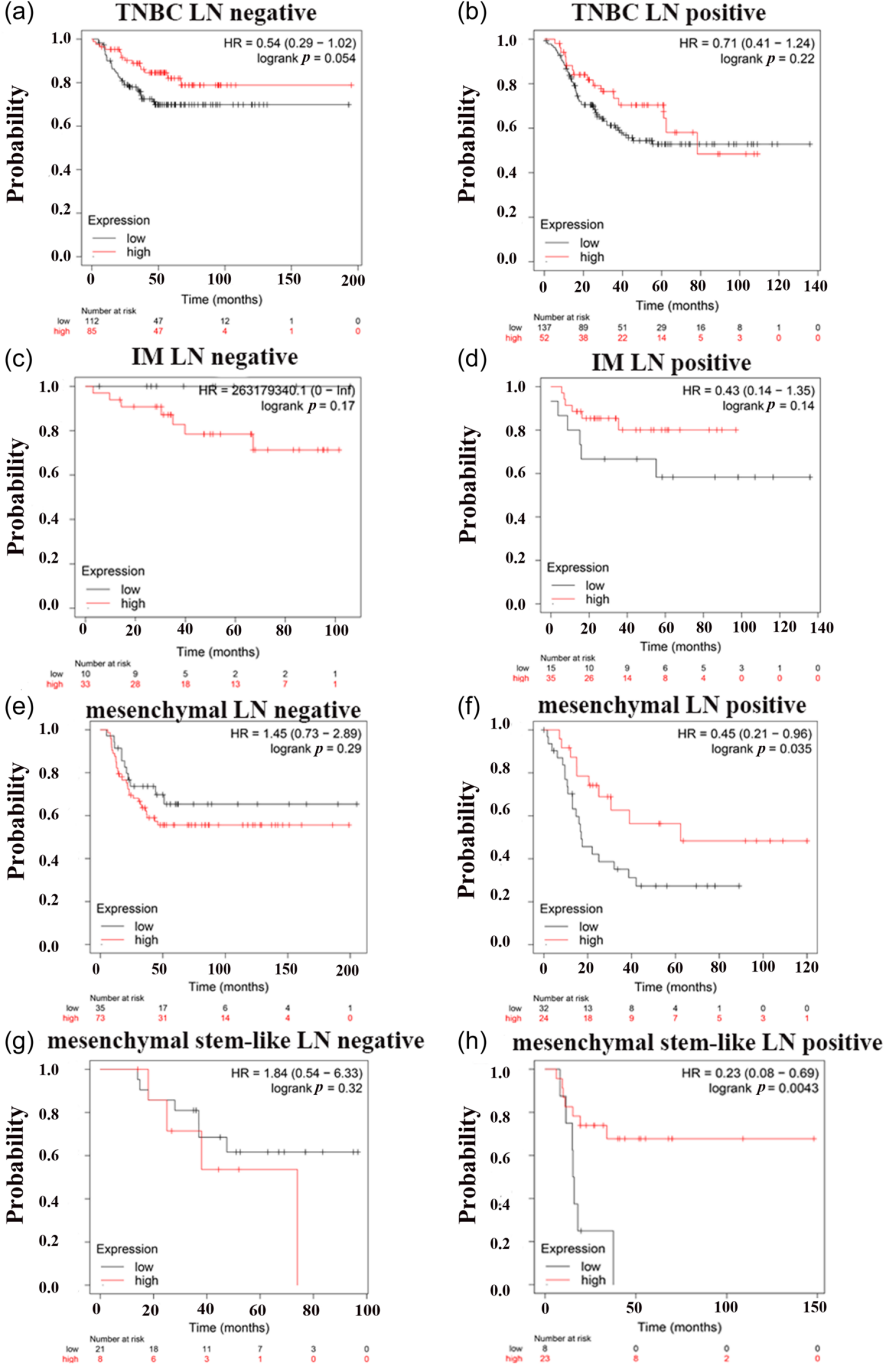
Kaplan‑Meier survival curve of patients with different subtypes of triple‐negative breast cancer (TNBC) and kallikrein 5 (*KLK5*) expression. (a and b) Kaplan‑Meier survival curve of TNBC with negative and positive lymph nodes (LNs) in accordance with *KLK5* expression. (c and d) Kaplan‑Meier survival curve of patients with the IM subtype of TNBC with negative and positive lymph nodes in accordance with *KLK5* expression. (e and f) Kaplan‑Meier survival curve of patients with the mesenchymal subtype of TNBC with negative and positive lymph nodes in accordance with *KLK5* expression. (g and h) Kaplan‑Meier survival curve of patients with the mesenchymal stem‐like subtype of TNBC with negative and positive lymph nodes in accordance with *KLK5* expression.

Relapse‐free survival (RFS) was chosen as the indicator of prognosis. High *KLK5* expression significantly correlated with worse RFS in breast cancer with lymph node (LN) positivity, suggesting that downregulation of *KLK5* may have no clinical significance in breast cancer without accurate subtyping (Supporting Information: Figure S1). We further analyzed the Kaplan‑Meier curves for subgroups of TNBC patients. There was no correlation between *KLK5* expression and RFS in either LN‐positive or LN‐negative TNBC cases (Figure 6). Thus, the prognostic significance of *KLK5* needs to be analyzed in different TNBC subtypes. Metaplastic TNBC samples in GSE165407 were classified as immunomodulatory (IM), mesenchymal (M), and mesenchymal stem‐like (MSL) subtypes. We next analyzed subgroups of LN status in these subtypes. *KLK5* was significantly associated with RFS in the M and MSL subtypes with LN positivity, which was consistent with our bioinformatic analysis that *KLK5* was downregulated in metaplastic TNBC. However, *KLK5* did not show an association with RFS in the IM subtype. We speculate that the M and MSL subtypes may be driven by EMT‐related pathways, so *KLK5* expression affects tumor prognosis, while the IM subtype is mainly driven by IM mechanisms, so *KLK5* does not have an influence. Together, these findings indicate that *KLK5* may serve as a biomarker in metaplastic TNBC with the M and MSL subtypes, especially with LN positivity.

### 3.6 *KLK5*‑associated gene analysis between *KLK5*‐high and *KLK5*‐low breast cancer patients

**Figure 7:**
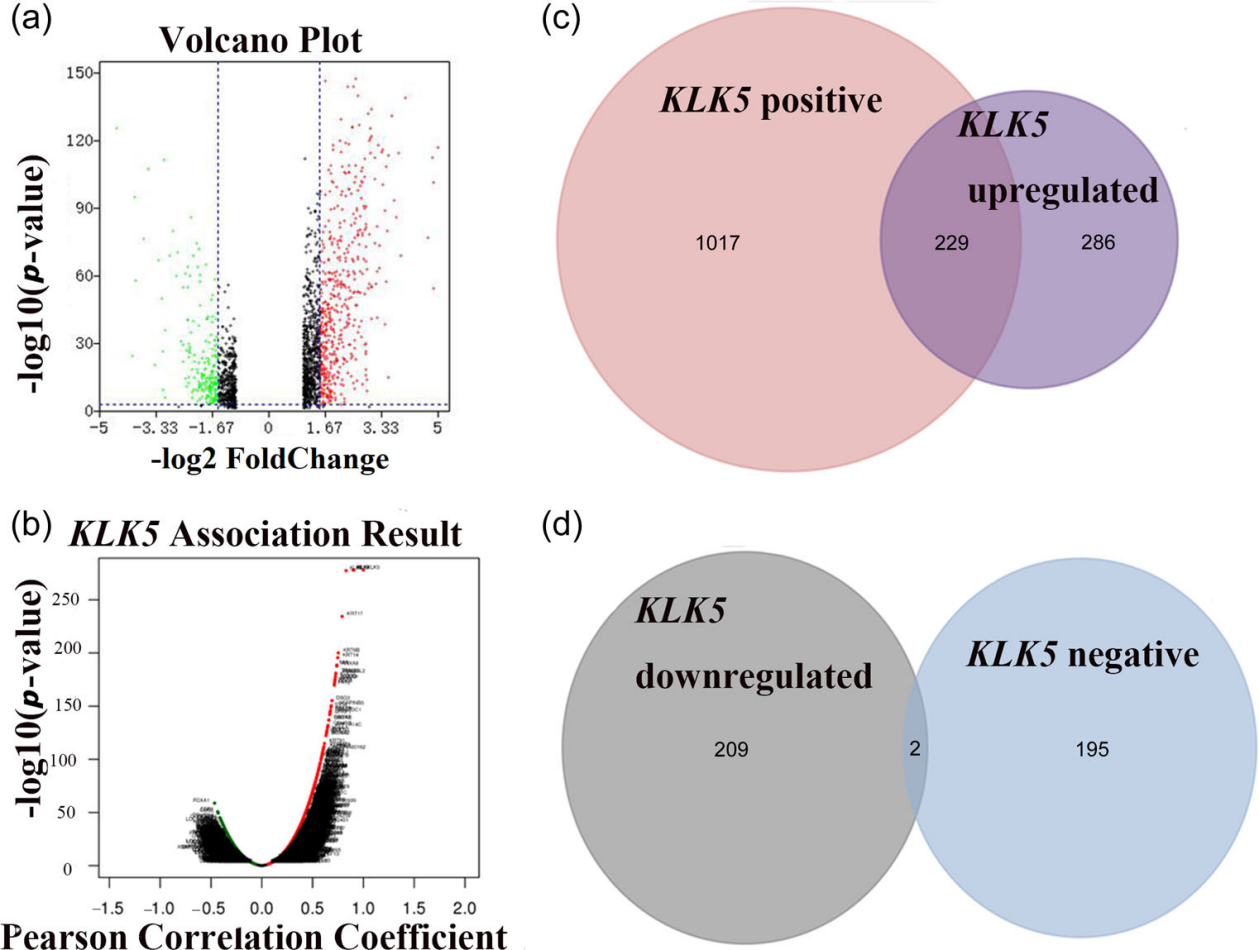
Genome‐wide genes associated with kallikrein 5 (*KLK5*) expression. (a) Volcano plot of different gene expression profiles between the *KLK5*‐low and *KLK5*‐high groups. (b) Volcano plots for the analysis of the co‐expression genes associated with *KLK5* expression using the LinkedOmics. (c) Overlapping genes between positively correlated genes and significantly increased genes. (d) Overlapping genes between negatively correlated genes and significantly reduced genes.

To further explore the role of *KLK5* in breast cancer, we compared the transcriptomes of the *KLK5*‐high and *KLK5*‐low groups using TCGA database. A total of 726 DEGs between the *KLK5*‐low and *KLK5*‐high groups (*p* ≤ 0.001, |log(FC)| ≥ 1.5; Supporting Information: Table S4) were screened; 515 genes were significantly upregulated and 211 genes were downregulated in the *KLK5*‐high group (Figure 7a). Next, we analyzed the co‐expressed genes of *KLK5* using the LinkedOmics database. A total of 1443 co‐expressed genes were significantly correlated with *KLK5* in breast cancer (FDR ≤ 0.001, *p* ≤ 0.001, and |person correlation| ≥ 0.3; Supporting Information: Table S5) (Figure 7b). A total of 1246 genes positively correlated with *KLK5* expression, and 197 genes negatively correlated with *KLK5* in breast cancer.

There were 231 overlapping genes identified by comparison of the DEGs between the significantly correlated genes in Supporting Information: Table S5 and significantly different genes in Supporting Information: Table S4 of *KLK5* (Supporting Information: Table S6), including 229 upregulated genes and two downregulated genes (Figure 7c,d).

### 3.7 Hub gene selection and analysis of overlapping genes

**Figure 8:**
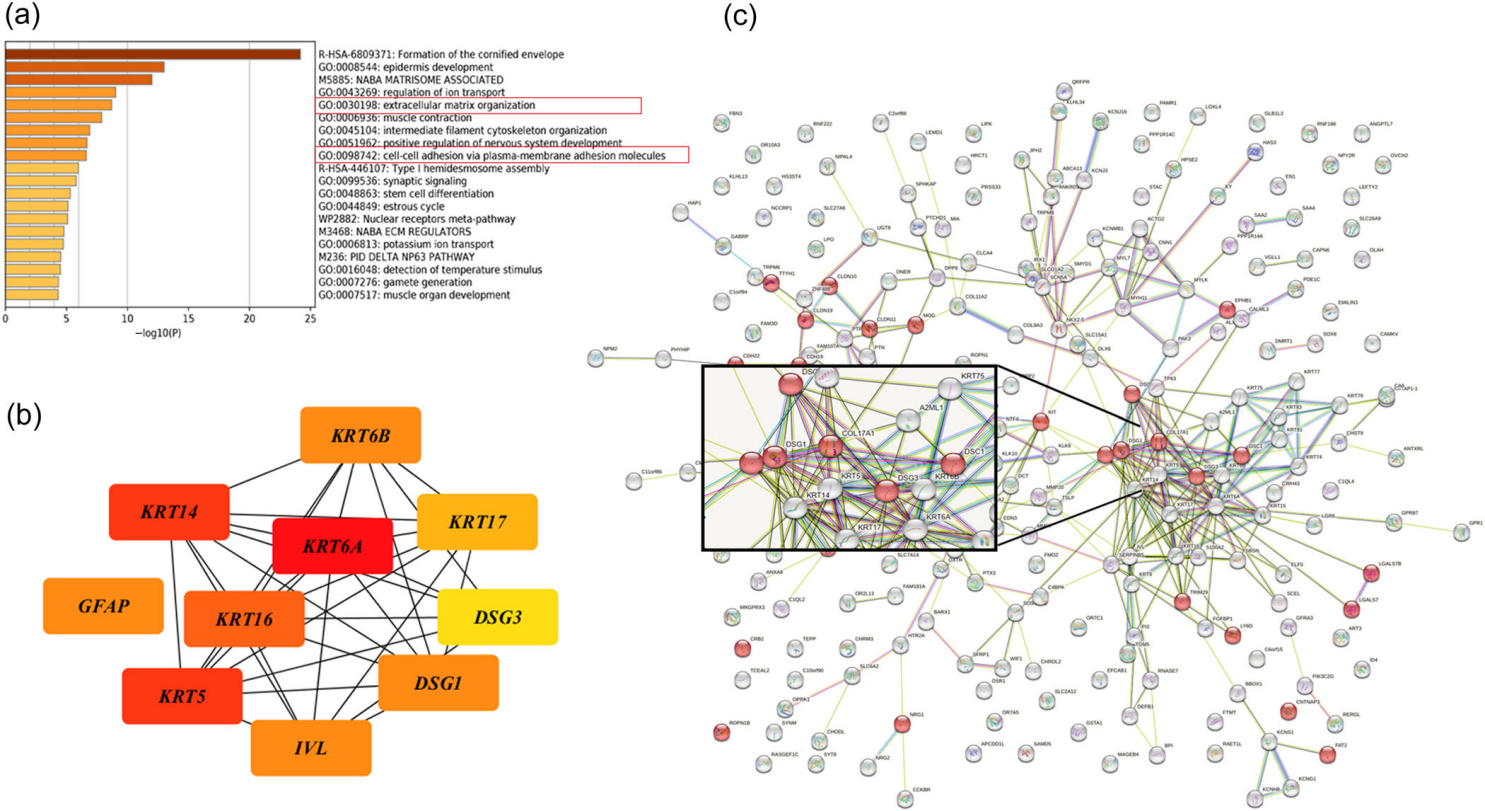
Enrichment of functions and signaling pathways of the overlapping genes in breast cancer. (a) Analysis of Gene Ontology (GO) and Kyoto Encyclopedia of Genes and Genomes (KEGG) pathways associated with kallikrein 5 (*KLK5*) expression. (b) Ten hub genes were screened using the protein–protein interaction network by Cytoscape software. (c) Visualization of 26 genes correlated with cell adhesion function by analyzing the STRING (Search Tool for the Retrieval of Interacting Genes) database.

To investigate the biological function of *KLK5* in breast cancer, KEGG and GO annotation of the 231 overlapping genes was performed using Metascape. Among the 20 top clusters of enriched sets, extracellular matrix organization (GO:0030198) and cell‐cell adhesion via plasma‐membrane adhesion molecules (GO:0098742) were directly correlated with EMT, which indicated *KLK5* was possibly involved in EMT in breast cancer (Figure 8a). To explore the core genes associated with *KLK5* involved in EMT, the top 10 hub genes (*KRT6A, KRT5, KRT14, KRT16, KRT6B, GFAP, IVL, DSG1, KRT17*, and *DSG3* genes) were screened out according to the node degree from cytoHubba software in Cytoscape (Figure 8b) and are listed in Supporting Information: Table S7. Among these genes, *DSG1* and *DSG3* are components of intercellular desmosome junctions, which mediate cell‐cell adhesion and correlate with EMT in cancer [25, 26]. We used the STRING database to analyze the PPI networks of the 231 overlapping genes. GO analysis showed that 26 genes were involved in cell adhesion function, including *DSG1* and *DSG3* (Figure 8c). These findings indicate that *DSG1, DSG3*, and *KLK5* are closely related genes and might be involved in EMT in metastatic TNBC and promote tumor progression.

## 4 DISCUSSION

MBC is a rare breast cancer subtype. Most of the MBCs are TNBCs and are poorly responsive to systemic therapy. Few diagnostic and prognostic markers for metaplastic TNBC and nonmetaplastic TNBC have been discovered.

Our bioinformatics analysis indicates that *KLK5* may be a pivotal molecule that plays a key role in the mechanism of tumorigenesis in metaplastic TNBC but not in nonmetaplastic TNBC. *KLK5* is a secreted serine protease encoded by the *KLK5* gene located on chromosome 19q13.4 [27]. *KLK5* is mainly present in the human epidermis and is involved in skin desquamation; *KLK5* is also found in other organs, including the breast, ovary, testis, vagina, and esophagus [28, 29, 30]. In our study, *KLK5* showed low expression in breast cancer but had no significant clinical value in breast cancer without precise molecular subtypes. Low *KLK5* expression indicated a worse clinical prognosis in metaplastic TNBC, but high *KLK5* expression in nonmetaplastic TNBC had no effect on clinical survival. The role of *KLK5* in breast cancer remains controversial. Downregulation of *KLK5* expression levels was observed in breast cancer specimens compared with benign specimens and exhibited a significant and independent value for the discrimination of malignant from benign mammary gland biopsies in logistic regression and receiver‐operating curve analysis [31]. Another study showed that enhanced signaling involving the oncogene *GNA13* downregulates *KLK5* gene transcription, which promotes breast cancer progression [32]. Moreover, reactivation of *KLK5* not only suppresses key EMT genes in breast cancer but also suppresses the mevalonate pathway [33]. High *KLK5* levels in serum were detected by enzyme‐linked immunosorbent analysis in some patients with ovarian (69%) and breast (49%) cancer [34]. The differences in *KLK5* serum and tissue levels suggest that *KLK5* may be regulated by a negative feedback mechanism in breast tissue to maintain the balance of the KLK proteolytic enzyme cascade.

*KLK5* plays a different role in different cancers. *KLK5* overexpression predicts a worse prognosis in various cancers, including cutaneous squamous cell carcinoma, colorectal adenocarcinoma, bladder tumors, and ovarian cancer, acting as an oncogene [35, 36, 37, 38]. *KLK5* acts as a tumor suppressor in hormone‐dependent tumors, such as prostate cancer, vaginal cancer, and breast cancer [30, 39, 40]. Our analysis suggests that EMT is the pathogenic mechanism by which *KLK5* functions in metaplastic TNBC. Additionally, *DSG1* and *DSG3* genes were identified as *KLK5* co‐expressed genes. *DSG1* and *DSG3* are components of intercellular desmosome junctions, which mediate cell‐cell adhesion and correlate with EMT in cancer progression. Therefore, the interaction between *KLK5, DSG1*, and *DSG3* may be an important mechanism for the development of metaplastic TNBC. Persistently elevated *KLK5* could induce the degradation of *DSG1*, and *DSG1* may cause the compensatory upregulation of *DSG3* [41, 42]. The mechanism by which *KLK5, DSG1*, and *DSG3* mediate EMT in metaplastic TNBC requires further exploration.

High *KLK5* expression was not found to play a significant role in nonmetaplastic TNBC. Our analysis showed that the main pathogenic pathway in nonmetaplastic TNBC was associated with abnormal inactivation of the ER signaling pathway. As TNBC lacks ER expression, the ER signaling pathway itself is not a direct pathogenesis pathway for TNBC, but altering the ERα‐dependent genes caused by ER pathway inhibition is an important mechanism for the rapid progression of TNBC. Nevertheless, many questions remain as to how *KLK5* is regulated in MBC by the mechanisms differing from its high expression in TNBC. According to research on the quantitative proteomic landscape of MBC relative to TNBC, the proteome of MBC has a highly enriched EMT phenotype, and MBC expresses proteins involved in the EMT process, which may contribute to a more stem‐like and aggressive phenotype than TNBC [43]. These results are consistent with our study, suggesting that *KLK5* is closely related to EMT progression. We did not find differential *KLK5* expression, which may be related to the small sample size, but we found high expression of the upstream factor GNA13 [32], which directly leads to a decrease in *KLK5* expression and indirectly supports our hypothesis. The tumor inhibition effect of *KLK5* in metaplastic TNBC and its relationship with clinical characteristics need to be verified with a large number of clinical specimens. Overall, the role of *KLK5* in metaplastic TNBC is important. *KLK5* may inhibit EMT through different mechanisms during tumor progression in metaplastic TNBC, which needs further exploration.

## 5 CONCLUSIONS

In conclusion, *KLK5*, which is differentially expressed in metaplastic TNBC and nonmetaplastic TNBC, may be a novel and independent biomarker for predicting the prognosis of metaplastic TNBC. Moreover, *KLK5* may be a driver gene in metaplastic TNBC because its low expression may affect the aggressiveness of metaplastic TNBC by promoting EMT. Further studies are needed to clarify the involvement of *KLK5* in EMT progression in metaplastic TNBC.

## AUTHOR CONTRIBUTIONS

**Yue Song**: Data curation (lead); formal analysis (lead); visualization (lead); writing—original draft (lead). **Guiying Bai**: Conceptualization (supporting); visualization (supporting). **Xiaoqing Li**: Methodology (equal); software (equal). **Liyan Zhou**: Formal analysis (equal); software (equal); visualization (equal). **Yiran Si**: Formal analysis (equal); methodology (equal); writing—review and editing (equal). **Xiaohui Liu**: Conceptualization (equal); methodology (equal). **Yilin Deng**: Software (equal); visualization (equal); writing—review and editing (equal). **Yehui Shi**: Supervision (equal); validation (equal).

## CONFLICT OF INTEREST STATEMENT

The authors declare no conflict of interest.

## ETHICS STATEMENT

This study was approved by an Institutional Review Board of Tianjin Medical University Cancer Institute and Hospital (Approval number: Ek2020008).

## INFORMED CONSENT

Tissue samples were collected from patients who provided informed consent.

## Supporting information

Supporting information.

Supporting information.

Supporting information.

## Data Availability

All data generated or analyzed on the basis of public databases in this study were obtained from the databases mentioned in the methods, and relevant descriptions and website links are included in this article. Independent cohort experimental data can be obtained from the corresponding author upon reasonable request.
