# Supplementary figures and images for: Bioinformatics analysis of human kallikrein 5 (KLK5) expression in metaplastic triple‐negative breast cancer
Source: Cancer Innov. 2023 Oct 15;2(5):376–90. doi: 10.1002/cai2.96 (PMC10686124; doi:10.1002/cai2.96)

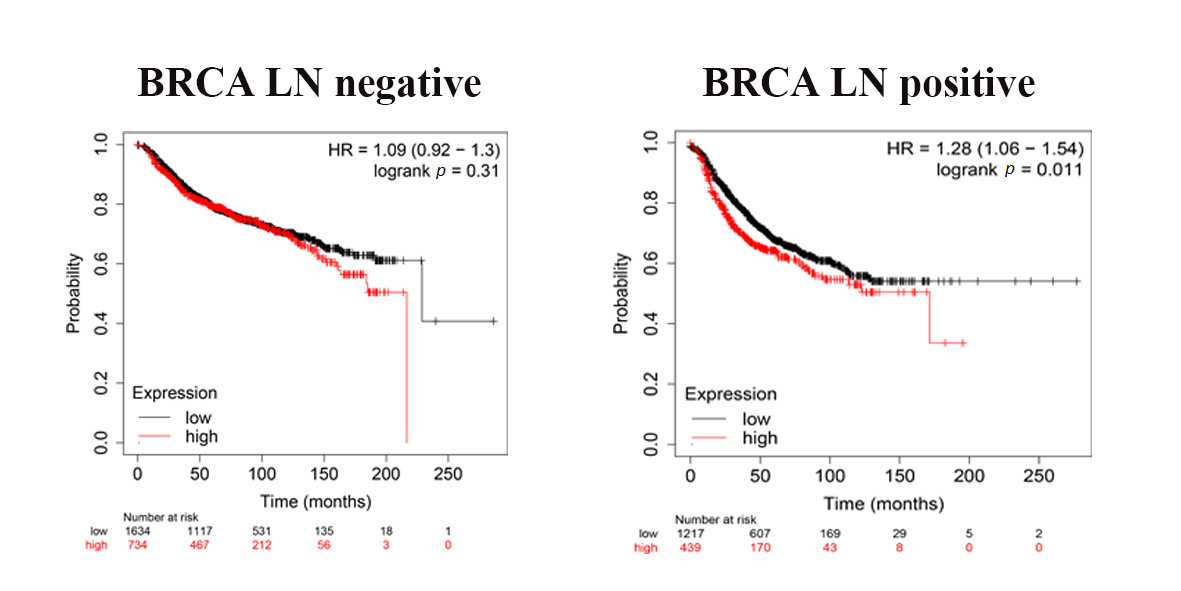

Supplement: Supplementary file 1 — Supporting information. [file CAI2-2-376-s003.tif]
