# Supplementary material for: Bioinformatics analysis of human kallikrein 5 (KLK5) expression in metaplastic triple‐negative breast cancer
Source: Cancer Innov. 2023 Oct 15;2(5):376–90. doi: 10.1002/cai2.96 (PMC10686124; doi:10.1002/cai2.96)
Supplement: Supplementary file 3 — Supporting information. [file CAI2-2-376-s001.docx]

**Figure S1** Kaplan-Meier survival curve of patients with BRCA and KLK5 expression.
